# Cochlear shape distinguishes southern African early hominin taxa with unique auditory ecologies

**DOI:** 10.1038/s41598-021-96543-w

**Published:** 2021-08-23

**Authors:** J. Braga, C. Samir, A. Fradi, Y. Feunteun, K. Jakata, V. A. Zimmer, B. Zipfel, J. F. Thackeray, M. Macé, B. A. Wood, F. E. Grine

**Affiliations:** 1 Centre d’Anthropobiologie et de Génomique de Toulouse, Université Paul Sabatier Toulouse III, Faculté de Médecine Purpan, 37 allées Jules Guesde, Toulouse, France; 2 Evolutionary Studies Institute, University of the Witwatersrand, PO WITS, Johannesburg, 2050 South Africa; 3 LIMOS, UMR 6158 CNRS-Université Clermont Auvergne, 63173 Aubière, France; 4 Faculty of Informatics, Technical University of Munich, Munich, Germany; 5 Véto 31, 73 Avenue du Général de Gaulle, 47000 Agen, France; 6 Center for the Advanced Study of Human Paleobiology, George Washington University, Washington, DC 20052 USA; 7 Department of Anthropology, Stony Brook University, Stony Brook, NY 11794 USA; 8 Department of Anatomical Sciences, Stony Brook University, Stony Brook, NY 11794 USA

**Keywords:** Evolutionary ecology, Palaeoecology, Evolutionary ecology, Palaeoecology, Evolution, Palaeontology, Taxonomy

## Abstract

Insights into potential differences among the bony labyrinths of Plio-Pleistocene hominins may inform their evolutionary histories and sensory ecologies. We use four recently-discovered bony labyrinths from the site of Kromdraai to significantly expand the sample for *Paranthropus robustus*. Diffeomorphometry, which provides detailed information about cochlear shape, reveals size-independent differences in cochlear shape between *P. robustus* and *Australopithecus africanus* that exceed those among modern humans and the African apes. The cochlea of *P. robustus* is distinctive and relatively invariant, whereas cochlear shape in *A. africanus* is more variable, resembles that of early *Homo*, and shows a degree of morphological polymorphism comparable to that evinced by modern species. The curvature of the *P. robustus* cochlea is uniquely derived and is consistent with enhanced sensitivity to low-frequency sounds. Combined with evidence for selection, our findings suggest that sound perception shaped distinct ecological adaptations among southern African early hominins.

## Introduction

The morphology of the bony labyrinth reflects the shape of its membranous compartments, whose receptors detect sound in the cochlea, and sense head position and motion in the vestibule and semi-circular canals, respectively^1–3^. The developmental stability^4^ and species-specific features^5–7^ of the bony labyrinth, and the relationships between cochlear morphology and auditory perception among mammalian species^8–10^, suggest that cochlear shape has the potential to provide evidence about the sensory ecology of extinct hominin taxa. Previous investigations have not detected significant cochlear differences among Plio-Pleistocene australopiths^1,2,11^. However, the complex shape of the cochlea, comprising a curve of decreasing radius that approximates but does not conform precisely to a logarithmic spiral with different degrees of torsion along its length, does not lend itself to being captured by the linear methods and 3D geometric morphometric (3DGM) approaches that were previously employed.

**Figure 1:**
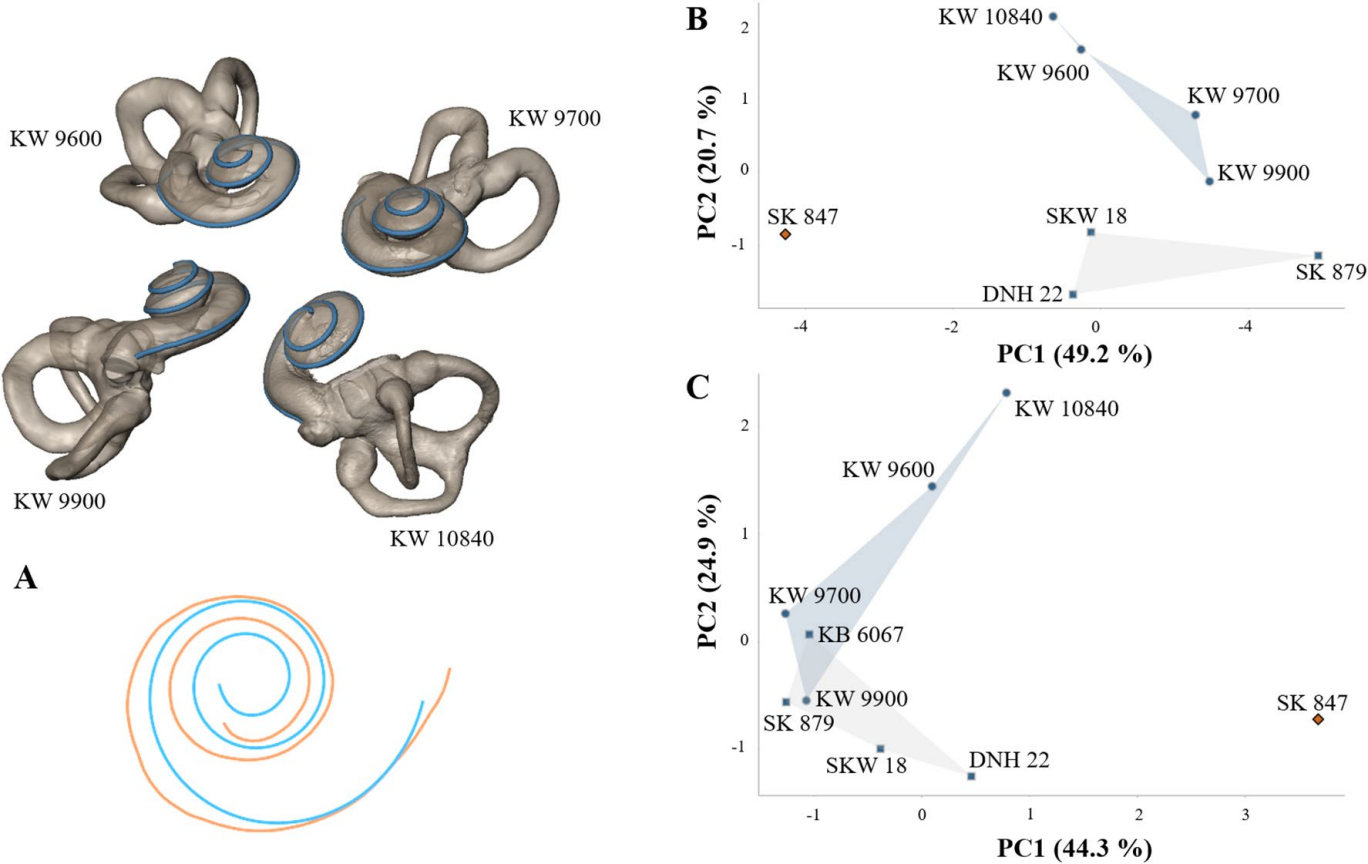
Four new fossil hominins recovered from Kromdraai (South Africa) attributed to *Paranthropus robustus*, and mean cochlear shape in this species*.* The new specimens from Kromdraai represent three juvenile (KW 9600, KW 9700 and 10840) and one adult (KW 9990) individuals (blue circles). Blue squares represent other *P. robustus* specimens from Kromdraai, Swartkrans and Drimolen. Only SK 847 is attributed to early *Homo* (orange diamond). (**A**) Fréchet mean computed from our *P. robustus* sample (blue) aligned with the cochlear curve of the SK 847 early *Homo* specimen (orange) using exactly the same reference system and orientation. (**B**,**C**) Two distinct principal components analyses (PCA, with the variance percentages indicated for PC1 and PC2) using (**B**) one data set with the two angles and the seven indices listed in Table S3 (the variables that contribute most significantly to PC1 are, with decreasing loadings: TLI, HZL/ANL, ECL/POL, APA < LSCm and ECL/ANL); (**C**) the previous data set adding the incomplete bony labyrinth of KB 6067 (with its damaged anterior semi-circular canal) from Kromdraai^27^ and excluding the HZL/ANL, POL/ANL and ECL/ANL indices (the variables that contribute most significantly to PC1 are, with decreasing loadings: COs < LSCm and TLI).

**Figure 2:**
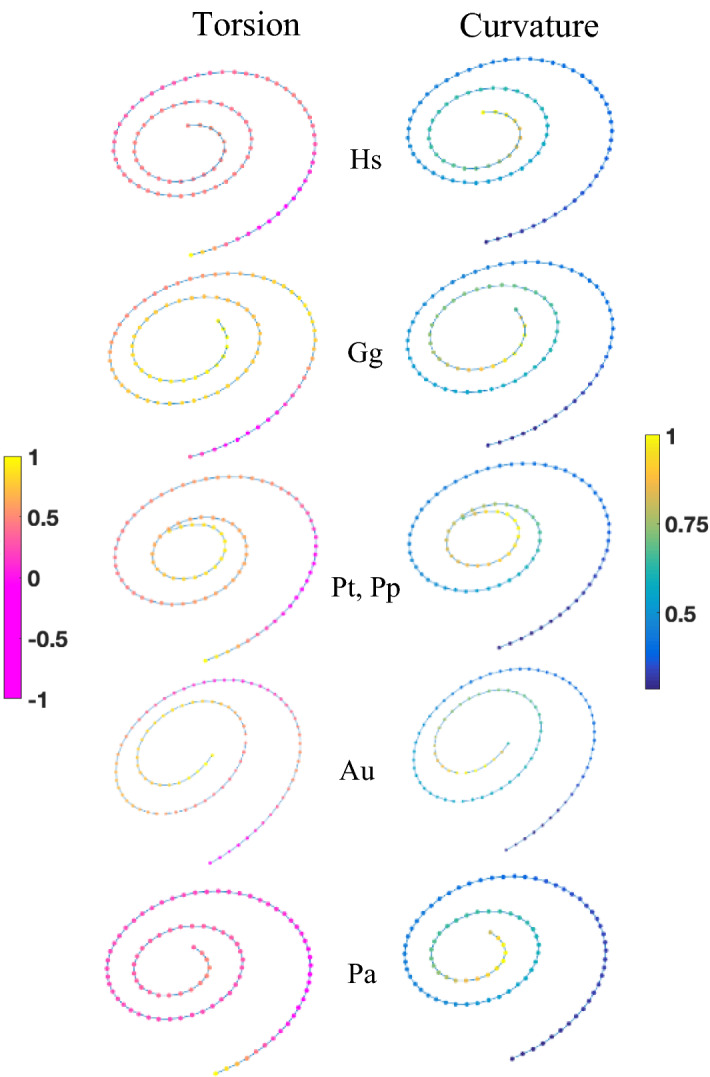
Curvature and torsion of mean cochlear shapes. Mapping curvature (blue to yellow) and torsion (purple to yellow) changes along the Fréchet means from basal to apical locations. *Hs* *Homo sapiens*, *Gg* *Gorilla gorilla*, *Pp* *Pan paniscus*, *Pt* *Pan troglodytes*, *Au* *Australopithecus*, *Pa* *Paranthropus*.

Here, we used diffeomorphometry^12–14^, which has detected sex-based and size-independent cochlear differences in modern humans^15^, to investigate potential differences in cochlear morphology among fossil hominin taxa from southern Africa. We analyze cochlear shape using a non-linear geometric framework that employs Fréchet means to define the optimal size-independent differences between complex shapes (“Methods”). The Fréchet mean^12,13^ of two points along a curved surface is located at equal geodesic distances (arc lengths) between them, whereas the standard arithmetic mean utilized in 3DGM analyses is located at mid-linear distance, and therefore lies outside the curve. This non-linear geometric framework, unlike 3DGM, allows measurements of local geometric properties^16^, including mapping changes in curvature (bending) and torsion (twisting) at any location along the length of the cochlea, from its base to the apex (“Methods”). We applied this method to micro-CT images of the cochlea of samples of museum specimens representing modern humans (*Homo sapiens*, n = 16), common (*Pan troglodytes*, n = 16) and pygmy (*Pan paniscus*, n = 16) chimpanzees, gorillas (*Gorilla gorilla*, n = 15), and Plio-Pleistocene hominin fossils from southern African cave sites belonging to *Australopithecus africanus* (n = 9)*, Paranthropus robustus* (n = 8) and undoubted early *Homo* (n = 1) (Tables S1,2). The Fréchet mean of *P. robustus* cochlear shape is illustrated in Fig. 1. The Fréchet means and curvature and torsion maps for each of the five genera (*Australopithecus*, *Paranthropus*, *Homo*, *Pan* and *Gorilla*) in our sample are illustrated in Fig. 2.

While most researchers agree that the australopith remains from Sterkfontein represent a single taxon, *A. africanus*^17,18^, others have suggested they sample two species of *Australopithecus*, one of which more closely “approaches” the morphology of *Paranthropus*^19^, but this latter suggestion has yet to convince most scientists^17,18,20–22^. The *A. africanus* bony labyrinth sample includes nine specimens from Sterkfontein, five of which (StW 252/255/259, StW 498, StW 504/505, StW 573 and StW 578) have been referred by one worker (Clarke^19^) to a second species with purported robust australopith affinities. There has been debate over the identification and specific attributions of early *Homo* fossils from Sterkfontein (specimens StW 53 and StW 151) and Swartkrans (specimen SK 847). While there is consensus that SK 847 belongs to early *Homo*^1,2^, the attribution of the two specimens from Sterkfontein is somewhat more contentious^23–25^, with some suggesting that they represent *A. africanus*. Here, we considered the StW 53 and StW 151 specimens as taxonomically indeterminate because of the disagreement relating to their assignation.

## Results

### Cochlear shape and early hominin taxonomy in southern Africa

The four recently-discovered bony labyrinths from Kromdraai (KW 9600, KW 9700, KW 9900 and KW 10840) included in this study (Fig. 1 and S1) considerably expand our knowledge of bony cochlear shape variation within *P. robustus*^20–22^. In a principal components analysis (PCA) of bony labyrinthine features, they and the other *P. robustus* specimens are well-separated from early *Homo* on PC1 (Fig. 1 and “Methods”). The features the new specimens from Kromdraai share with other *P. robustus* fossils, and which differentiate them from early *Homo*, include a smaller transverse labyrinthine index, a less inclined ampular line and a less inclined cochlear basal turn relative to the orientation of the horizontal semi-circular canal, plus larger ECL/POL and ECL/ANL indices (“Methods” and Table S3).

**Figure 3:**
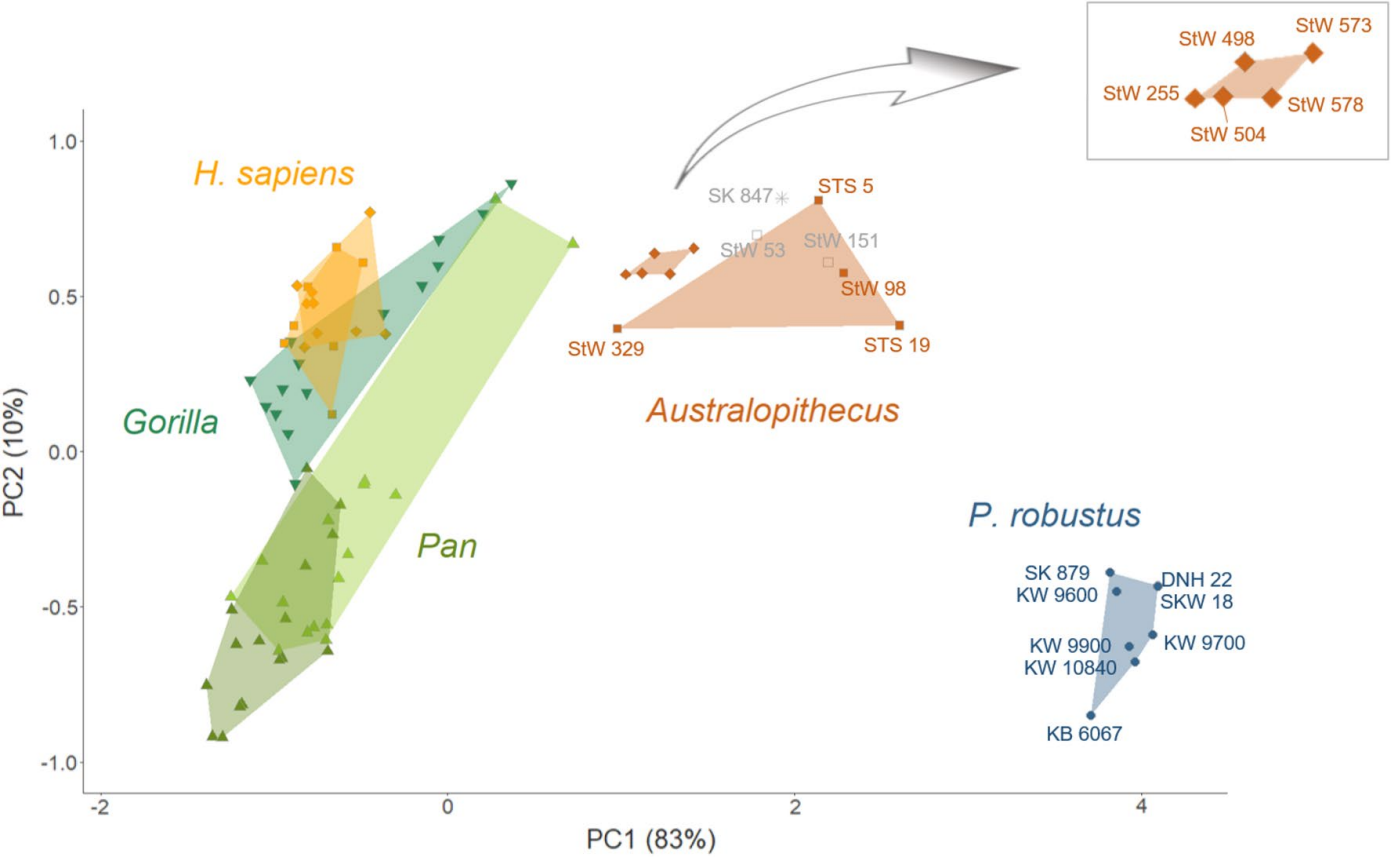
Tangent principal component analysis (TPCA) of cochlear curves compared using curvature and torsion. Blue circles are for *P. robustus* from Kromdraai (including the new specimens from Kromdraai, KW 9600, KW 9700, KW 9900, KW 10840), Swartkrans and Drimolen; brown diamonds and squares are for *A. africanus* from Sterkfontein, including five specimens that have been suggested sample a second species^19^ (diamonds) also illustrated in the enlarged box (top right); light and dark green triangles are for pygmy and common chimpanzees, respectively; green inverted triangles are for gorillas; orange squares and diamonds are for male and female modern humans, respectively; star is for SK 847 (early *Homo*); open grey squares are for StW 151 and StW 53 (here considered as “indeterminate”). Note that DNH 22 and SKW 18 overlap because their cochlear curves are nearly identical and their PC1 and PC2 values are similar.

We combined the juvenile and adult specimens in our samples because among modern humans, cochlear shape (assessed with diffeomorphometry) is age independent from birth^15^. In the PCA of bony labyrinthine features (with the first two PC scores), there is no difference between the three new juvenile (KW 9600, KW 9700, KW 10840) and the new adult (KW 9900) specimen from Kromdraai (Fig. 1), and nor are there any consistent differences between juveniles and adults in the combined *P. robustus* sample. There is also no consistent difference in cochlear shapes between juveniles and adults in the *A. africanus* sample (Fig. 3).

A PCA on the tangent space (TPCA)^13,15^, which summarizes most of the variation in cochlear shape (Fig. 3), yields better discrimination than 3DGM (Fig. S2). On PC 1, which captures c.83% of the variance, all seven *P. robustus* specimens form a tight cluster well away from any other group, although they overlap with chimpanzees on PC 2, which captures c.10% of the variance. *Australopithecus* specimens, which show a greater range of variation on PC 1, occupy an intermediate position between *P. robustus* and the extant taxa, particularly *H. sapiens* and *Gorilla*.

**Figure 4:**
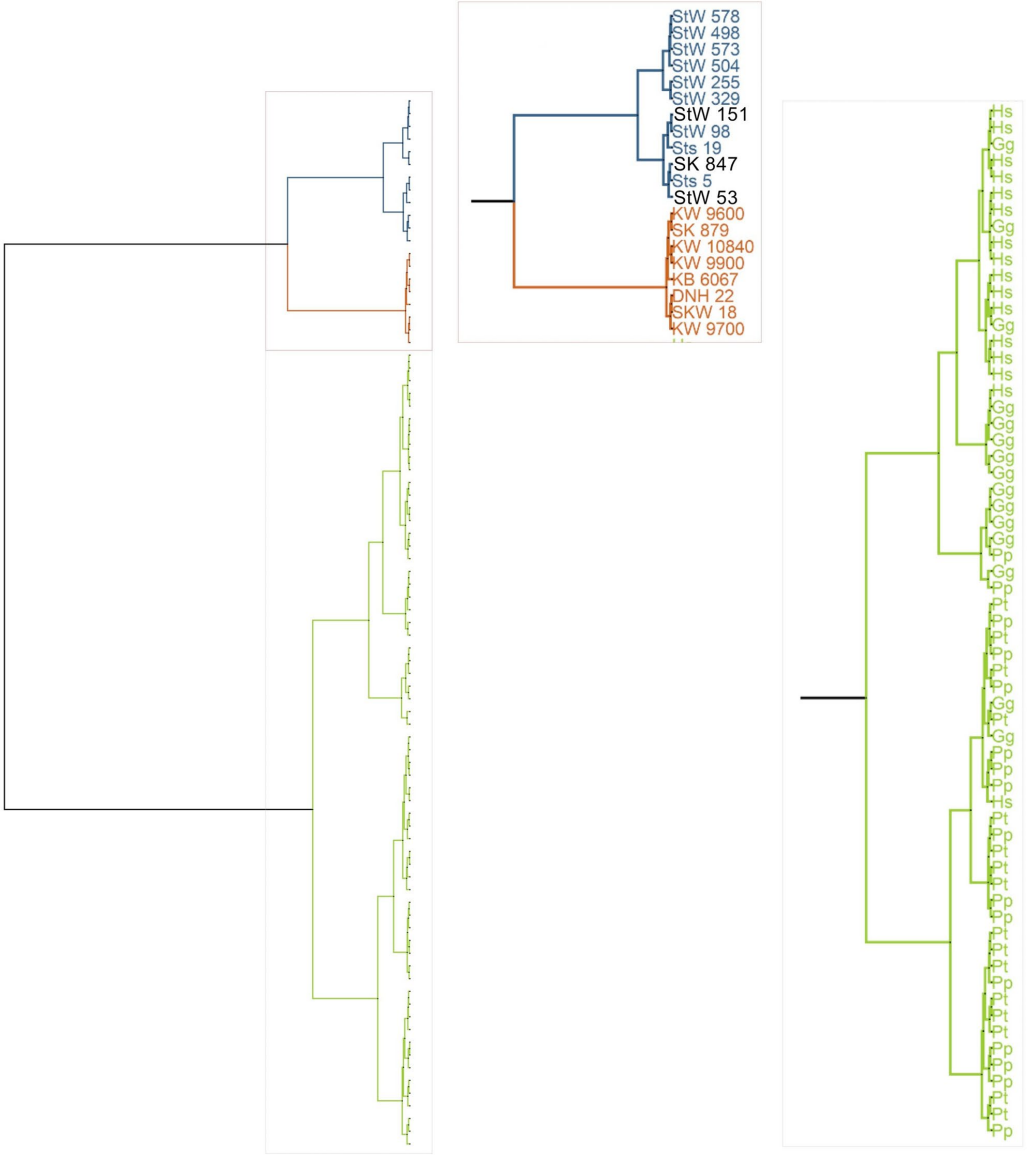
Assessments of the clustering performance of the TPCA. The best classification is achieved with three clusters representing extant species (green color, Hs, *Homo sapiens*; Gg, *Gorilla gorilla*; Pp, *Pan paniscus*; Pt, *Pan troglodytes*), *A. africanus* (blue color) and *P. robustus* (brown color). Note that the SK 847 (early *Homo*), StW 151 and StW 53 (here considered as ‘unknown’) are illustrated in black and cluster with *A. africanus* specimens.

Classification and clustering (evaluated by the balanced accuracy and the V-measure) using the first three PCs (97% of the total variance) of the TPCA (“Methods”) results in three clusters comprising, respectively, (i) extant species, (ii) *Australopithecus* and early *Homo*, and (iii) *P. robustus* (Fig. 4). In one cluster, the *P. robustus* specimens show an exceptionally low level of diversity. In the second cluster, the five specimens from Sterkfontein that have been suggested to represent a second species^19^ are not separated from the four other *A. africanus* fossils (i.e., they belong to the same cluster as all other *A. africanus* fossils, and show close similarities to the StW 329 *A. africanus* specimen) (Fig. 4), and they are less *P. robustus*-like than other specimens (Sts 5, Sts 19 and StW 98) from the site (Fig. 3). When compared to the variability in cochlear morphology within modern humans, chimpanzees or gorillas, the *Australopithecus* sample from Sterkfontein does not show more variation (Figs. 3 and 4). This result is consistent with the hypothesis that the cochlear variability sampled within the *Australopithecus* sample from Sterkfontein can be accommodated within a single species^18^. Here, too, there is no evidence of an age effect, with the younger StW 98, and the older StW 329 and StW 255 fossils grouping together within the *Australopithecus* sample. While it could be argued that our results confirm the australopith affinities of StW 53 and StW 151 (Figs. 3, 4), it should be noted that the cochlear shape of SK 847 is also similar to that of the *A. africanus* specimen Sts 5 (“Mrs. Ples”) as well as to StW 53. When compared with the variation among *Pan, Gorilla* and *Homo sapiens*, the different cochlear morphologies of *P. robustus* and *A. africanus* are consistent with their generic distinction.

When compared to *A. africanus, P. robustus* is distinctive in that at any location, from the base to the apex, it shows a lower torsion along its Fréchet mean (Fig. 2). This difference is particularly noticeable towards the cochlea’s last turn where the lowest-frequency waves are resolved^8,9^. Near the cochlear apex, the *P. robustus* Fréchet mean suggests a near absence of torsion and a high degree of curvature, which results in a hook-like shape.

### Effects of selection and phylogeny on cochlear shape

To assess whether the morphological differences between *P. robustus* and *A. africanus* were more likely to be the result of neutral evolution or directional selection^27^, we compared the pattern of maximal between-group variance relative to within-group variance (“Methods”)^28,29^. Directional selection assumes a deviation from proportionality between the covariance matrix of related taxa means (B) and the pooled within-taxa covariance matrix (a weighted average of the taxa covariance matrices as an estimate of the ancestral covariance matrix) (W). A principal coordinate analysis of the covariance matrices of TPCA values shows that the within-taxa matrices form a relatively homogeneous cluster around their weighted average W, whereas B falls far outside this cluster (Fig. S3). A significant deviation from proportionality (p-value < 0.001) between (B) and (W) separates *P. robustus* from the other taxa. This result, together with a relative eigenanalysis indicating that the inter-taxa covariance exceeds the variation expected for neutral evolution, is consistent with cochlear shape having evolved under directional selection in *P. robustus*.

Because the Fréchet mean similarities between more closely-related species are no greater than those between more distantly-related taxa (i.e., there is no (B)/t relationship), variation in cochlear shape did not increase linearly with evolutionary time, as would be expected in the case of a Brownian-motion model of evolution (“Methods”)^30^. We therefore used the alternative Ornstein–Uhlenbeck (OU) model that is most suitable for selection^30^, and applied it to the PC1 scores from the TPCA by using two alternative scenarios of rates of cochlear shape change. It is noteworthy that there was a rapid divergence between the *A. africanus* and *P. robustus* lineages between 3 and 2 Ma, whereas early *Homo* retained a more ancestral condition than *P. robustus* (Fig. S4).

## Discussion

Neither linear measurements nor 3DGM morphometric techniques have been able to discern clear-cut differences between *A. africanus*, *P. robustus* and early *Homo* with respect to their middle ear ossicles^31^, bony labyrinths^1,2,11^ or cochleae^2,11^. The present study using diffeomorphometry has shown that the cochlear shapes of both *A. africanus* and especially *P. robustus* are distinct from those of modern humans and the African apes. We find genus-level differences between *Paranthropus* and *Australopithecus* in southern Africa, but no cogent evidence for a taxonomic distinction within the *A. africanus* sample from Sterkfontein. In light of the variation documented here in *A. africanus*, morphological diversity is low in *P. robustus* as sampled at Kromdraai, Swartkrans and Drimolen (Figs. 3, 4). The KNN classification and V-measure from a TPCA of cochlear shape highlights the near similarity between specimens from Swartkrans (SKW 18) and Drimolen (DNH 22) and the absence of morphological change that may be related to time with the *P. robustus* lineage. Thus, evidence from the bony labyrinth is not consistent with microevolution in *P. robustus*^21^, although different results from other regions of the skeleton would perhaps not be surprising.

Mosaic evolution is also evident within the bony labyrinth. For example, with respect to SK 847 and StW 53, while “extreme” differences in their semicircular canal proportions suggest that it is “highly unlikely” they belong to the same early *Homo* species^1^, their cochlear morphology is remarkably similar.

Recent analyses suggest that *Homo* and *Paranthropus* are sister clades, and Bayesian inference has shown high levels of support for *A. africanus* being the sister taxon to the *Homo* + *Paranthropus* clade^32^. Thus, the similarity in cochlear shape between SK 847 (early *Homo*), StW 53 and StW 151 (here treated as taxonomically indeterminate) and *A. africanus* suggests that the cochlear morphology of southern African early *Homo* was more conservative compared to *P. robustus*. Our analyses reveal strong selection for cochlear morphology in *P. robustus*, and suggest that selection occurred early in the evolutionary history of this species. Stable isotope (δ^13^C) data and faunal analyses indicate that in southern Africa both *P. robustus* and early *Homo* inhabited more open environments than *A. africanus*^33^. If one posits the co-occurrence of early *Homo* and *P. robustus,* the relatively rapid divergence of *P. robustus* cochlear shape from the ancestral condition (Fig. S4) could have resulted in acoustic niche partitioning between these two southern African hominin lineages.

Micromechanical models^34^ consistently suggest that an increased cochlear curvature enhances low-frequency (LF) sensitivity in the apical portion where curvature is the greatest^35,36^. Given our finding of significant size-independent and significant differences in cochlear mean shapes between species, and the unique hook-like shape (i.e., near absence of torsion and high degree of curvature) near the apex in *P. robustus*, cochlear micromechanics^34–36^ suggest enhanced LF sensitivity in this species. Comparative studies over a range of primates indicate a relationship between LF sensitivity and frequency difference limen (FDL) sensitivity^37^, wherein frequency resolution increases linearly as frequency increases. This enhanced capacity to distinguish between two pure tones would allow individuals to more reliably discriminate species-specific communication signals. Compared to *Pan troglodytes*, modern humans show a wider auditory band of maximum sensitivity that emphasizes higher frequencies^38,39^ and higher frequency resolution^40^ that would facilitate short-distance calling. In contrast, the cochlear morphology of *P. robustus* (Fig. 2) suggests an emphasis on higher frequency resolution in the lower frequency range. One possible explanation is that *P. robustus* evolved a novel auditory specific mate recognition system (SMRS). The ability to rapidly discriminate conspecific communication signals is well known in primates^40,41^. Any such auditory SMRS may be influenced by the nature of the habitat in which it is used^41^, and it is possible that higher frequency resolution in the lower frequency range was required for the preferred habitat of *P. robustus*.

We recognize that it is not necessary to interpret all morphological differences in the context of adaptive functionality^42^, but the unique cochlear morphology of *P. robustus* is most likely to have been produced under strong directional selection (Fig. S3). Because the number of specimens (n), the dimensionality of the TPCA space (p), and the ratio n/p are important factors for covariance estimation, larger sample sizes would strengthen the statistical power of this analysis. Our results nevertheless suggest that the distinctive cochlear shape of *P. robustus* represents a unique auditory adaptation among southern African hominins. The phylogenetic results also lend support to the hypothesis that there was rapid, directional evolution of cochlear shape in *P. robustus.* The obvious question is whether this cochlear shape is autapomorphic for *P. robustus*, or whether it is shared by its congeners in eastern Africa. If it is, this would be compelling evidence for *Paranthropus* monophyly, although if it is not, this would not constitute evidence against monophyly. Of equal interest is whether the eastern African representatives of early *Homo* exhibit the same configuration as their congeners from southern Africa. Cochlear morphology has the potential to improve our understanding of the paleobiology of extinct hominin taxa.

## Methods

### Taxonomic attribution of the new fossil hominin specimens from Kromdraai

Among the four new fossil hominin specimens from Kromdraai (Text S1, Table S2, Fig. S1) one is an adult (KW 9900) and three are juveniles (KW 9600, KW 9700, KW 10840). The juvenile status was assessed by measuring the degree of opening of the subarcuate fossa^26,44,45^, a canal that extends through the arc of the anterior semi-circular canal and gradually obliterates during development. The small variation in the degree of fossa closure between KW 9600 (7.2%, mean of right and left sides), KW 9700 (6.4%, left side) and KW 10,840 (5.8%, mean of right and left sides) suggest that they represent three close-in-age juvenile individuals. Three other *P. robustus* specimens in our sample are also juveniles: (i) KB 6067 represents the youngest specimen as judged from the degree of its fossa closure^26^, (ii) SKW 18 is clearly associated with SK 52 to form a compound skull^46^, and (iii) TM 1517^43^. The two latter specimens show unerupted M3s with some root development internally visible from their micro-CTs (pers. obs). Three *Australopithecus* specimens also represent juvenile individuals. The higher degree of opening of the fossa in StW 98 (17.8%) suggest it is younger than StW 329 (5.8%) and StW 255 (4.5%). The juvenile status of StW 151 is confirmed by its opened fossa (5.2%). We investigated whether KW 9600, KW 9700, KW 9900 and KW 10840 showed more similarities to *P. robustus* from Kromdraai (TM 1517 and KB 6067), Swartkrans (SK 879 and SKW 18) and Drimolen (DNH 22), or to early *Homo* (SK 847). To do so, the micro-CT data were resliced in a plane that best fitted the horizontal semi-circular canal. One of us (JB) measured the transverse labyrinthine index (TLI), the inclination of the ampular line and the cochlear basal turn relative to the orientation of the horizontal (or lateral) semi-circular canal (APA < LSCm and COs < LSCm, respectively)^47^. Six additional indices of the bony labyrinth were added: the arc lengths of the horizontal (HZ), posterior (PO) and anterior (AN) semi-circular canals (noted HZL, POL and ANL, respectively) measured by placing a curve along their outer circumference. We then computed the HZL/ANL, HZL/POL and POL/ANL indices. The length of the cochlea (ECL^26^) was also size-standardized by HZL, POL and ANL and provided three additional indices (ECL/HZL, ECL/POL and ECL/ANL). We ensured the best-possible measurements (taken with the Avizo software package, www.vsg3d.com/avizo) by using simultaneously the resliced stacks of micro-CT images and the 3D reconstruction of the bony labyrinths. In order to assess potential measurement errors, each variable was measured twice on five randomly selected bony labyrinths with more than a one-day interval between each trial. The intraclass correlation coefficient results for the reproducibility of the measurements showed good agreement (ICC > 0.8). In order to assess the compatibility between our measurements of APA < LSCm, COs < LSCm and TLI and the ones made on the same specimens from medical CTs^47^, we compared the two sets of measurements for TM 1517, SK 879 and SK 847 (Table S3). We observed identical or very similar values. The bony labyrinth of the type specimen of *P. robustus* from Kromdraai (TM 1517)^43^ can be measured only partly (Text S2) due to precipitated crystallized material within the bone that prevents the accurate measure of cochlear shape.

### Ethical approval

All the steps of the present study were performed in accordance with relevant guidelines and regulations. No data used in this study involved experimentation, risk or constraint added by the research. Only museum specimens were included in this study. We obtained permissions to access these museum specimens that have already been used in several published studies^2,15,26^.

### Cochlear shape: maps of curvature and torsion

The use of 3DGM assumes that cochlear curves are linear (or Euclidian) spaces, whereas they are not. Non-linear methods are needed to analyze cochlear pure shape^12,13^ in 3D. Therefore, we used diffeomorphometry, a method based on the uniform scaling and nonlinear (elastic) registration of 3D non-linear spaces (called “Riemannian manifolds”)^12,13^ that has been applied in evolutionary anthropology^48,49^. Its efficacy for taxonomic discrimination of fossil hominin teeth has been compared to results obtained with 3DGM^14^. The 3DGM cannot capture key geometrical properties of 3D cochlear curves, with their two most important properties: the curvature (bending) and the torsion (twisting) that can be defined intuitively as follows. Curvature describes the degree of bending at any location along the cochlear curve from the base to the apex. The greater the curvature, the faster is the change of its tangent vector direction. Torsion describes how the 3D curve twists out the plane of curvature. The curvature and torsion were calculated (with Matlab 2016a) by using the Frenet-Serret formulae^50^ that can be described intuitively as follows. A curve can be regarded as the path of a moving particle. We measure its bending by using the curvature as the rate of change for the unit tangent vector (normalized first derivative). We also measure how quickly the curve twists by using the rate of change of the oscillating plane by the tangent vector and the normal vector. We emphasize here that our mapping of the changes in curvature along the 3D cochlear curve (Fig. 2) is fundamentally different from the global assessment of curvature represented by only one measure such as the “radii ratio”^2,9^, which is the ratio of the radii of the basal and apical termini of the cochlear curve. Assessments of “radii ratio” obtained from photographs^51^ embed the cochlear curve within a plane and, therefore, cannot capture 3D shapes and torsion and cannot be compared with our measurements. Moreover, since “radii ratio” overlap between *P. robustus* and *A. africanus*, as well as between species with larger (e.g., gorillas) and smaller (e.g., pygmy chimpanzees) skull bases^2^, the global curvature of the cochlea is not higher when this organ is “packed” in smaller cranial bases.

### Cochlear curve: sampling and analysis

After the segmentation of each bony labyrinth from its micro-CT volumes, we placed the cochlear curve as already described^2^ and we resampled it to 200 semi-landmarks. In order to assess potential errors in the placement of the curves, we selected randomly ten cochleae. One of us (JB) placed the curves twice on each cochlea with more than a one-week interval between each session. The significance of Procrustes distances within and between the two repeated measurements was assessed via permutation tests (1000 random permutations). They indicated that the differences in shape between specimens were significantly higher (at p < 0.01) than those within repeated measurements. To compute elastic deformations between two corresponding cochlear curves, we adapted the general shape analysis framework^16^. These deformations are generated using vector fields between curves under an elastic Riemannian metric (Text S3). The computations on Riemannian manifolds and the TPCA were performed in Matlab 2016a by using the packages ‘SRVF_FDA’^16^ and ‘ROPTLIB’^52^. The TPCA can also be performed by using the R package ‘shapes’^53^.

### Assessment of the clustering performance of the TPCA results

We performed K-nearest neighbour (KNN) classification (implemented in the R package ‘yardstick’^54^) in which each specimen in the sample is assigned a class (here, a taxon) depending on its similarities with its neighbours in TPCA space (Text S4). This method considers imbalanced class sizes to account for underrepresentation of small samples classes. The performance of this classification is defined as the average of the true positive rates of each class. To evaluate the clustering, we use the V-measure^55^ (implemented in the R package ‘sabre’^56^), a metric that is independent of the size of the data, the number of classes, and the number of clusters (i.e., the clustering).

### Analysis of Procrustes coordinates of landmark data

We performed a generalized Procrustes superimposition of the landmark coordinates and a PCA with the R packages ‘Morpho v2.8’^57^ and ‘geomorph v3.3.2’^58^). We did not use between-groups PCA (bgPCA) because of the problems that have been noted when using fewer groups than variables^59^. Moreover, because of fundamental differences between 3DGM and our approach based on diffeomorphisms (see above), we expect that bgPCA applied on Procrustes coordinates will not improve the delineation of fossil taxa.

### Comparison of variance–covariance patterns of differences within and between taxa

Relative eigenanalysis^28^ was done with the ‘vcvComp’ R package^29^. We used the three first PCs of the TPCA results (83%, 10% and 4% of variance represented by PC1, PV2 and PC3, respectively) and six taxa to perform this analysis. Indeed, when we evaluated the clustering performance of our TPCA, we concluded that all the *Australopithecus* specimens in our sample were more likely grouped in one species: *A. africanus*. The maximum likelihood (ML) test suggested a deviation from proportionality between B and W (p = 0.00106) and thus the action of selective forces. Since this test does not specify the magnitude of deviation from proportionality, we performed an ordination of the covariance matrices, together with W and B. Relative to the heterogeneity of taxa covariance matrices, B clearly deviates from W along the first principal coordinate (Fig. S3) and we reject the null hypothesis that the matrices are proportional to each other. The covariance matrices of gorillas, chimpanzees and modern humans group close together, in close proximity to W. The covariance matrices of *P. robustus*, *A. africanus* and specimens attributed to early *Homo* deviate from this pattern, with *P. robustus* being an outlier. A relative PCA of B with respect to W provides the following three successive eigenvalues: 44.79, 5.89 and 0.24. The first relative eigenvalue is more than 7 times larger than the second one, which is significant at p < 0.001, and similarly for the last relative eigenvalue. The first and second relative eigenvalues (44.79 and 5.89) largely exceed the threshold estimated on the basis of genetic data by using the ${F}_{ST}$ test^60^. ${F}_{ST}$ represents the fraction of genetic diversity attributable to between-group differences. Under pure genetic drift, ${F}_{ST}$ values among extant humans and African apes range between 0.09 to 0.42 within species, and between 0.49 to 0.94 among species^61,62^.

### Ornstein–Uhlenbeck (OU) process

Since we did not find a relationship between (B) and evolutionary time since divergence (t) (as expected in Brownian motion), we fitted a OU process to the TPCA scores on PC1. We used a phylogeny that combines a calibration with phylogenomics^63^ and fossil evidence fixing the ages of some internal branches, nodes and tips^64^. Because the phylogenetic position of *A. africanus* and *P. robustus* is not resolved, we simulated the changes of TPCA scores on PC1 along each of two alternative scenarios (Fig. S4): (i) an evolutionary continuity between *A. africanus* and *P. robustus*, the latter being considered as a sister clade of *Homo* with a MRCA set at 2.8 Ma; (ii) *P. robustus* and *Homo* with a MRCA set at 3.5 Ma and with *A. africanus* considered as a sister group to both. The optimal hyper-parameter was estimated with Markov-Chain Monte Carlo (MCMC) simulations from a maximum total of 5.000.000 iterations.

## Supplementary Information

Supplementary Information.

## Data Availability

The datasets generated during and/or analyzed during the current study are available from the corresponding author on request. Some datasets and codes supporting the current study have not yet been deposited in a public repository because they are part of further investigation.
