# Supplementary material for: Cochlear shape distinguishes southern African early hominin taxa with unique auditory ecologies
Source: Sci Rep. 2021 Aug 23;11:17018. doi: 10.1038/s41598-021-96543-w (PMC8382707; doi:10.1038/s41598-021-96543-w)
Supplement: Supplementary file 1 — Supplementary Information. [file 41598_2021_96543_MOESM1_ESM.docx]

Supporting Information for

**Cochlear shape distinguishes southern African early hominin taxa with unique auditory ecologies**

J. Braga^1,2*^, C. Samir^3^, A. Fradi^3^, Y. Feunteun^3^, K. Jakata^2^, V.A. Zimmer^4^, B. Zipfel^2^, J.F. Thackeray^2^, M. Macé^5^, B.A. Wood^6^, F.E. Grine^7,8^

**1**. Centre d’Anthropobiologie et de Génomique de Toulouse, Université Paul Sabatier Toulouse III, Faculté de Médecine Purpan, 37 allées Jules Guesde, Toulouse, France.

**2**. Evolutionary Studies Institute, University of the Witwatersrand, PO WITS, Johannesburg, 2050, South Africa.

**3**. LIMOS, UMR 6158 CNRS-Université Clermont Auvergne, 63173 Aubière, France.

**4**. School of Biomedical Engineering & Imaging Sciences, King’s College London, London, United Kingdom.

**5**. 73 Avenue du Général de Gaulle, 47000 Agen, France.

**6**. Center for the Advanced Study of Human Paleobiology, George Washington University, Washington DC 20052, USA.

**7**. Department of Anthropology, Stony Brook University, Stony Brook, NY 11794, USA.

**8**. Department of Anatomical Sciences, Stony Brook University, Stony Brook, NY 11794, USA.

* Corresponding author : [jose.braga@univ-tlse3.fr](mailto:jose.braga@univ-tlse3.fr)

**Contents of this file**

Texts S1-S4

Figures S1-S4

Tables S1-S3

**Text S1**

**Context of the new fossil hominin specimens from Kromdraai**

The fossil hominin temporal bones newly used in this study belong to four specimens (KW 9600, KW 9700, KW 9900, KW 10840) and were recovered in situ from Unit P at Kromdraai (Table S2). Unit P represents one of the two newly recognized hominin-bearing sedimentary deposits that have been identified during recent fieldwork at this site (Braga et al., 2017). Unit P was previously identified as “Member 2” when it was considered as “sterile” (Vrba, 1981). It was still named “Member 2” in the most recent description of the stratigraphy of Kromdraai (Bruxelles et al., 2016) and when the discovery of new fossil hominin specimens was subsequently announced (Braga et al., 2017). The term “Unit” was recently used to designate Unit P and any other depositional interval distinguishable above and below at the scale of the Kromdraai site (Ngoloyi et al., 2020). This change was decided to follow the conventional lithostratigraphic terminology used by the International Commission on Stratigraphy (stratigraphy.org), in which a “Member” does not designate “the smallest formal unit in the hierarchy of sedimentary lithostratigraphic units”. The stratigraphic sequence at Kromdraai starts with Unit A which contains no fossils and corresponds to the “Stony breccia” as described in Brain (1958), and “Member 1” as described in Vrba (1981), Partridge (1982) and Bruxelles et al. (2016). Unit P is stratigraphically older than the immediately overlying sedimentary deposits of “Member 3” - as described in Vrba (1981), Partridge (1982) and Bruxelles et al. (2016) - and subsequently subdivided by Bruxelles et al. (2016) into “Member 3” (now renamed Unit Q) and its overlying “Submember 4.1” (now renamed Unit R). A few hominin specimens were recovered within Units Q-R either in situ or ex-situ (Braga et al., 2017). Before the present study, only two non-dental diagnostic cranial remains were available in the hominin assemblage from Kromdraai (Braga et al., 2013): TM 1517, the unprovenanced type specimen of *P. robustus* (Broom, 1938) and KB 6067, an isolated left petrous bone (Braga et al., 2013). As detailed in Braga et al. (2013), whereas KB 6067 can be tied to Units Q-R, TM 1517 comes from “significantly younger layers”. Therefore, the new hominin specimens used in the present study are stratigraphically older than both TM 1517 and KB 6067 specimens. The spatial patterning of the fossil hominin and non-hominin assemblage recovered in situ from Unit P until 2017 (Ngoloyi et al., 2020) describes four main clusters interpreted as either areas of higher density of fossils, or as accumulations resulting from particular processes (e.g., distinct entrances). Among the four hominin specimens from Kromdraai newly described in this study (Table S2), three (KW 9600, KW 9700 and KW 9900) were recovered in Cluster 3 (Ngoloyi et al., 2020). KW 10840 was not incorporated in the spatial analysis of the fossil assemblage from Unit P because it was discovered after the initiation of this study. We can nevertheless add that KW 10840 was found very close to the north-western boundary of Cluster 3 (Ngoloyi et al., 2020) at an elevation of 1471 m.

KW 9600 (Table S2, Fig. S1) represents a juvenile individual that preserves both right and left petrous temporal bones. The left petrous is better preserved. Most of the anterior and posterior surfaces of the petrous pyramid are preserved. The broken anterior and inferior surfaces expose the middle ear cavity with the oval window, and the prominence for the carotid canal. The angle between the anterior and posterior petrosal surfaces is less than 90° above the opening for the internal acoustic meatus. The superior petrosal margin becomes rounded at the level of the arcuate eminence, which is broad and well-developed. The posterior surface shows well-preserved apertures of the internal acoustic meatus and the petromastoid canal (i.e., the obliterated subarcuate fossa). The cochlear and vestibular aqueducts are slightly abraded at its inferior margin. On the right, a prominent flange of bone forms the superior margin of the openings for the vestibular aqueducts.

KW 9700 (Table S2, Fig. S1) consists of most of the petrous part, and part of the mastoid, parts of the left temporal bone of a juvenile individual. Most of the posterior endocranial surface of the petrous pyramid is well-preserved and meets at approximately a right angle with the posterior surface at the superior petrosal margin. The posterior margin of the specimen is the occipitomastoid suture. Further lateral on the posterior endocranial surface is a deep sigmoid sinus groove that is c.3.6 mm wide. The broken anterior and inferior surfaces expose a short section of the carotid canal and the middle ear cavity with the oval window. The posterior surface shows well-preserved apertures of the internal acoustic meatus, the petromastoid canal (i.e., the obliterated subarcuate fossa), and the openings for the vestibular aqueducts.

KW 9900 (Table S2, Fig. S1) represents an adult specimen that preserves the right temporal bone, and the petrous and mastoid parts of the left temporal bone. The right temporal bone provides a high level of detail for the tympanic plate and petrous. The anterior and posterior endocranial surfaces meet at less than a right angle in the region of the aperture for the internal acoustic meatus. The posterior surface also shows well-preserved apertures of the obliterated subarcuate fossa and the openings for the vestibular aqueducts that form a triangular flange. Posterolateral to the openings for the vestibular aqueducts, there is a pronounced and broad depression (cerebellar fossa). On the left side, where the occipitomastoid suture is preserved, the sigmoid sinus groove is c. 5.5 mm wide.

KW 10840 (Table S2, Fig. S1) represents a juvenile individual that preserves both temporal bones. The left temporal bone provides a high level of detail for the tympanic plate, mandibular fossa and petrous. The anterior and posterior endocranial surfaces meet at approximately a right angle above the opening for the internal acoustic meatus, but at less than a right angle above the cerebellar fossa. The arcuate eminence is well-developed. The posterior surface shows the well-preserved aperture of the subarcuate fossa and the openings for the vestibular aqueducts. On the posterior endocranial surface and further lateral to the occipitomastoid suture is an approximately 7.5 wide impression of the sigmoid sinus groove.

**Text S2**

**Taxonomic attribution of the new fossil hominin specimens from Kromdraai**

When bony labyrinths were too incomplete, such as in TM 1517, they were removed from the PCA. In this case, we compared too incomplete specimens by using the measurements listed in Table S2 separately. In most instances, the new hominin specimens from Unit P at Kromdraai show the greatest similarities to *P. robustus*, including its holotype (TM 1517). The four TLI values show a very limited range of variation (29°-31°) and fall well within the known range of variation of *P. robustus* (Z scores < 1), very close to the holotype value (TM 1517) and well below the SK 847 one. More variability among the four new specimens from Kromdraai is observed for APA<LSCm and COs<LSCm. The four APA<LSCm values fall within the known range of variation of *P. robustus* (Z scores < 1.5) and below the SK 847 value. When dealing with the COs<LSCm angle, the values of two out of the four new specimens from Kromdraai (KW 9700 and KW 9900) fall within the known range of variation of *P. robustus* (Z scores < 1) whereas the two other specimens (KW 9600 and 10840 with 51° and 52°, respectively) fall above the maximum value for *P. robustus* (44°) (with Z scores between 5 and 5.5). The Cos<LSCm angle in SK 847 is 64°-68°. It is therefore more likely that the KW 9600 and 10840 specimens expand the *P. robustus* range of variation of the Cos<LSCm angle.

**Text S3**

**Cochlear curve: sampling and analysis**

Curve registration, denoted $\hat{\nu}$, and comparisons are computed under a unified framework.

Let $c:\left[ 0,1 \right]\to\mathbb{R}^{3}$ be a cochlear open, parameterized curve. In this work, $c$ is represented by the function $\eta:\left[ 0,1 \right]\to\mathbb{R}^{3}$ defined as $\eta\left( t \right)=\frac{\dot{c}\left( t \right)}{\sqrt{|\dot{|c}(t)||}}\left( or 0 \right)$, where $\dot{c}$ is the derivative of $c$ and |$|\cdot||$ is the standard norm in $\mathbb{R}^{3}$. Because $\eta$ is defined using the derivative of $c$, translation is consequently removed in the analysis. However, the mapping $c\Leftrightarrow(c(0),\eta)$ is a bijection; thus, to bring translation back into the framework we store the starting point of each curve. Furthermore, rotation is removed using orthogonal Procrustes. The space of all $\eta$s is denoted by

$\mathcal{M=}\left\{ \eta:[0,1]\to\mathbb{R}^{3} \left| \int_{0}^{1} ||\eta(t){||}^{2}dt<\infty\right. \right\}$.

Because we seek a representation of curves that is invariant to reparameterization, this variability must be removed from the representation (Kendall, 1984; Srivastava and Klassen, 2016; Samir and Adouani, 2019). This is performed using the following formulation: Define $\mathcal{V}=\{\nu:\left[ 0,1 \right]\to\left[ 0,1 \right]|\nu\left( 0 \right)=0,\nu\left( 1 \right)=1,0<\dot{\nu}<\infty\}$ as the group of orientation preserving diffeomorphisms of $[0,1]$ (Kendall, 1984; Srivastava and Klassen, 2016) or a manifold of nonnegative cumulative distributions (Samir et al., 2019). For a curve $c$ and a function $\nu\in\mathcal{V}$, the transformed curve is given by $c\circ\nu$, and the corresponding $\eta$ of the transformed curve is $\left( \eta\circ\nu\right)\sqrt{\dot{\nu}}$. In order to unify all elements in $\mathcal{M}$ that denote the same curve, we define equivalence classes (also called orbits) of the type $\left[ \eta\right]=\{\left( \eta\circ\nu\right)\sqrt{\dot{\nu}}| \nu\in\mathcal{V}$}. The set of all equivalence classes is denoted by $\mathcal{S=M/V}$ and we are interested in computing geodesic paths and distances on this space. Then, a metric on $\mathcal{S}$ is needed to compare any two shapes; we impose the $\mathbb{L}^{2}$ metric, which turns out to be invariant to curve parameterizations. Furthermore, it can be shown that, under the $\eta$ representation, the $\mathbb{L}^{2}$ metric corresponds to an elastic metric on the original space of curves; in a pairwise comparison of curves, it allows bending and stretching/compressing of a curve to ensure optimal matching and deformation^16^. Such an optimal deformation between two shapes is given by the geodesic path, and the distance between them is given by the length of this path. The strength of this approach is that it not only provides a distance between curves, thus quantifying differences between them, but also a geodesic path between their corresponding shapes.

To see how this can be used in the problem at hand, consider two cochlear curves $c_{1}$ and $c_{2}$, represented by $\eta_{1}$ and $\eta_{2}$, respectively. In order to compute the geodesic between their equivalence classes $[\eta_{1}]$ and $[\eta_{2}]$, we fix $\eta_{1}$ and find the optimal reparametrization of $\eta_{2}$ by solving $\hat{\nu}=\text{arg}\text{inf}_{\nu\in\mathcal{V}}\parallel\eta_{1}-\left( \eta_{2}\circ\nu\right)\sqrt{\dot{\nu}}\parallel^{2}$. The function $\hat{\nu}$ provides the optimal correspondence of points across the two curves (Srivastava and Klassen, 2016). Furthermore, the energy used to compute this optimal registration is a proper distance. As a next step, we define $\eta_{2}^{*}=(\eta_{2}\circ\hat{\nu})\sqrt{\dot{\hat{\nu}}}$ and compute a geodesic path between $\eta_{1}$ and $\eta_{2}^{*}$ in $\mathcal{M}$. Since $\mathcal{M}$ is a vector space, the geodesic path between any two points is given by a straight line: $\beta\left( \tau\right)=\left( 1-\tau\right)\eta_{1}+\tau\eta_{2}^{*}$, where $\tau\in[0,1]$; $\beta$ is a geodesic path between the two given curves such that at $\tau=0$ it is in $[\eta_{1}]$ and at $\tau=1$ it is in $[\eta_{2}]$. In order to use these tools in our problem, we consider that the original curves have been represented with $\{\eta_{i}^{*}{\}}_{i=1}^{n}$, then their Fréchet mean is a minimizer of the variance (Srivastava and Klassen, 2016), satisfying

$$\mu=\text{arg}\text{min}_{\eta\in\mathcal{M}}\sum_{i=1}^{N} \text{inf}_{\nu_{i}}||\eta-\eta_{i}^{*}||^{2}.$$

**Text S4**

**Tangent principal component analysis (TPCA) of cochlear curves**

As the dimensionality of an observation or amount of data to be processed grows, there is a need to reduce the dimensionality in order to perform advanced statistical methods. To this end, PCA has been employed as the common technique on vector spaces. However, when the input data lie on a nonlinear manifold, which is the case here, a nonlinear generalization, usually called Tangent Principal Component Analysis (TPCA) is often employed (Dryden and Mardia, 1998). TPCA exploits the local linearization of the manifold: Compute the intrinsic mean of the data lying on the manifold and then map all observations to the tangent space with the help of the Riemannian Log map. Then it performs PCA on the tangent space and projects the principal vectors back on the manifold using the Riemannian exponential map (the inverse of the Log map). In order to make this method applicable in our context, we need to define the tangent space, the exponential map and its inverse at least in a local neighborhood. Therefore, the numerical solution should be computationally efficient and accurate.

Since $\mathcal{M}$ is a submanifold of the Hilbert sphere, at any curve $\eta\in\mathcal{M}$, we define the tangent space and we denote $T_{\eta}\mathcal{M}$. We equip the tangent space of $\mathcal{M}$ with a smoothly varying Riemannian metric that measures shortest lengths on the shape space. The metric defined on $\mathcal{M}$ has a nice physical interpretation in being an elastic metric allowing both bending and stretching. Another important step in our shape analysis is to compute geodesics between shapes with respect to the chosen metric. With respect to the $\eta$-function, $\mathcal{M}$ is represented as a submanifold of the Hilbert sphere and obviously lot is known about the geometry of a sphere, including geodesics, Log and exponential maps. Therefore, geodesics between any two elements $\eta_{1}$ and $\eta_{2}$ (not antipodal to $\eta_{1}$) on $\mathcal{M}$ are great circles. This formulation gives the geodesic passing through $\eta_{1}$ with a velocity vector $v$ at $t=0$. As a result, we have an explicit expression for the exponential map. The length of the geodesic determines an elastic quantitative distance between any two shapes $\eta_{1}$ and $\eta_{2}$ in $\mathcal{M}$. Conversely, given two shapes $\eta_{1}$ and $\eta_{2}$, the Log map allows the recovery of the tangent vector $v$ between them.


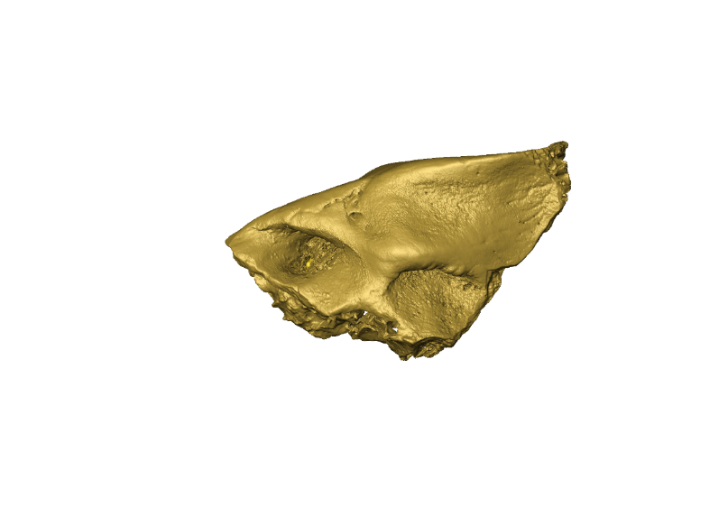

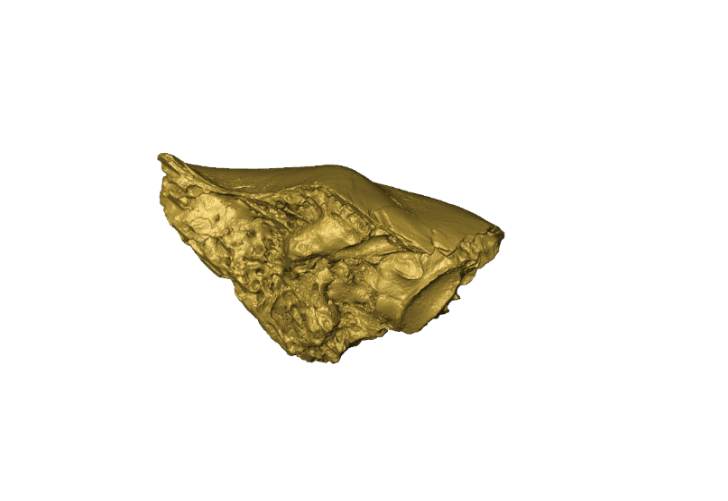

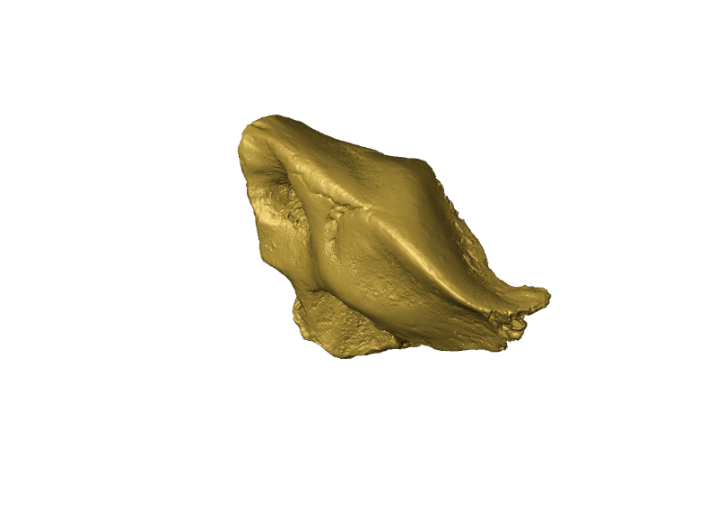

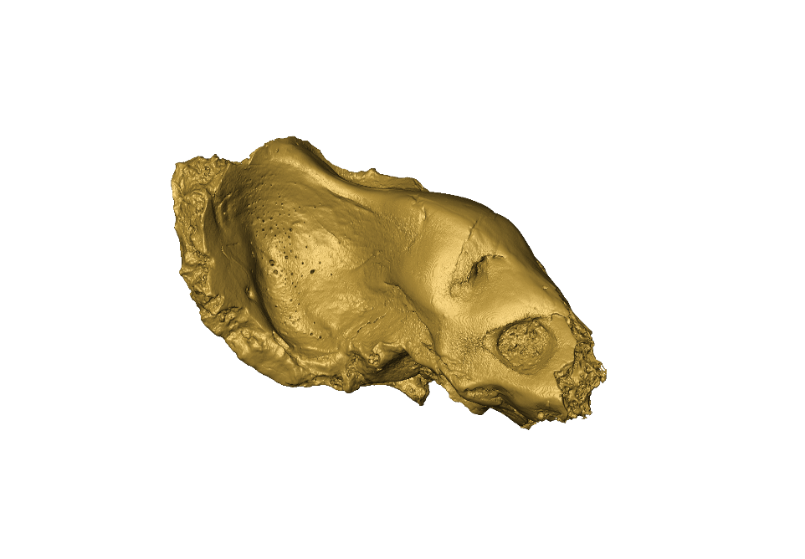

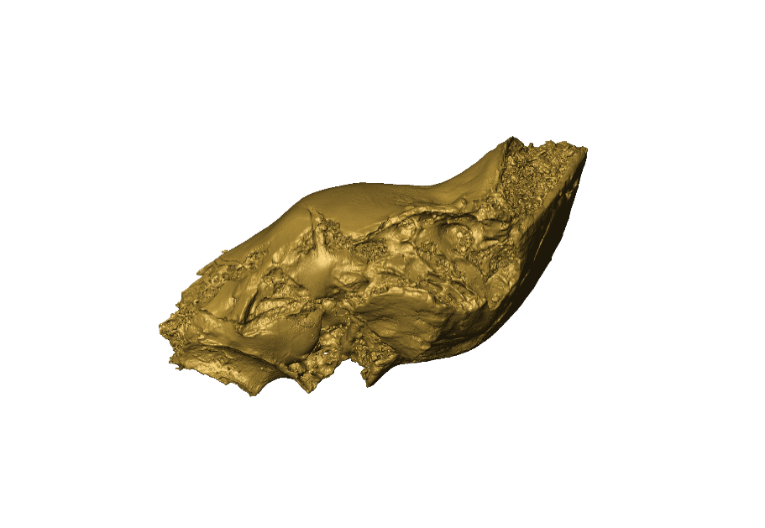

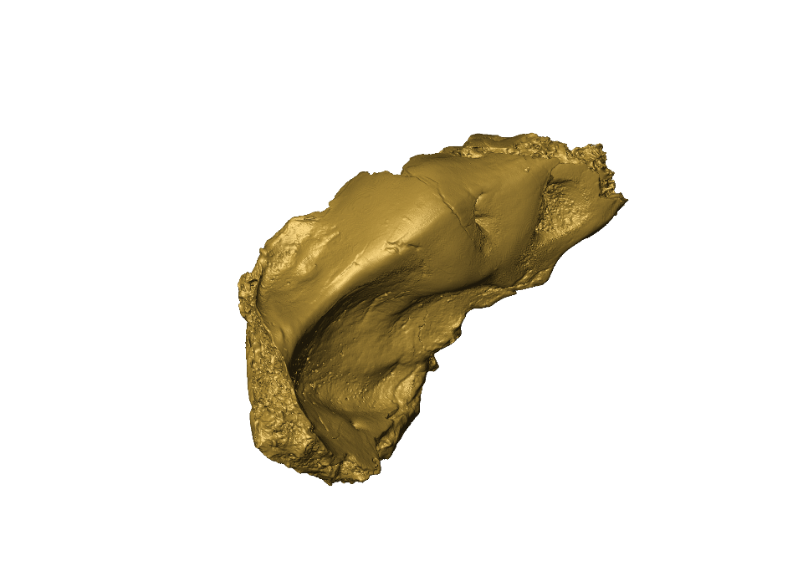

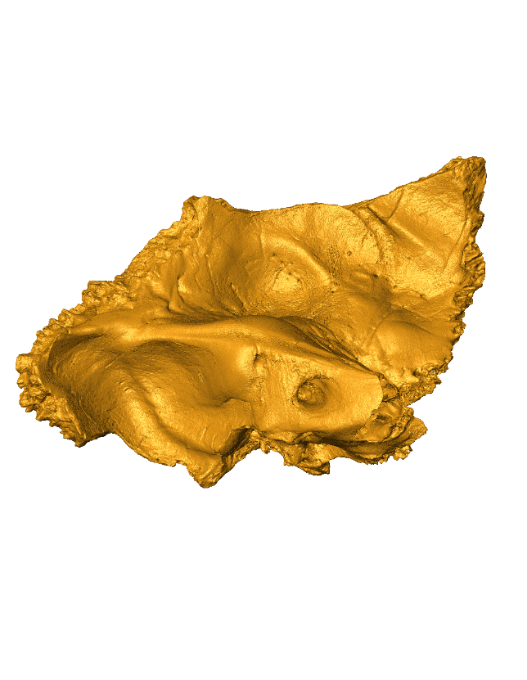

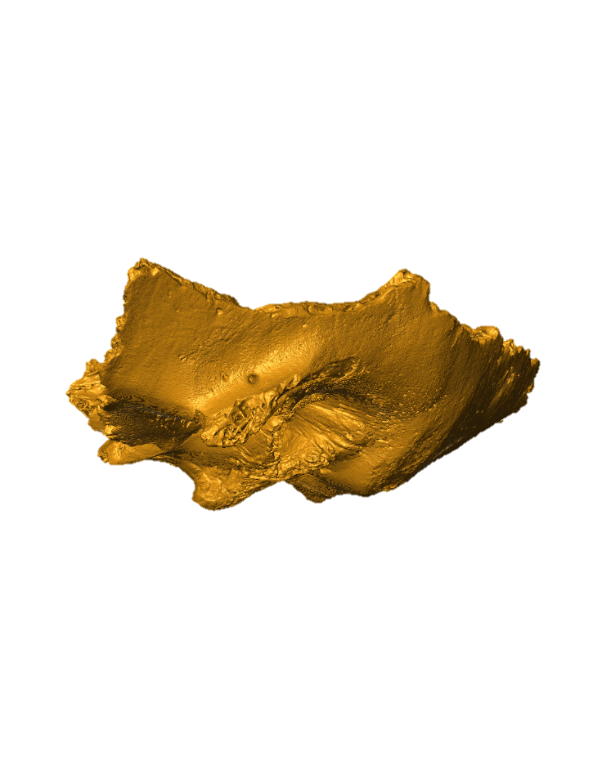

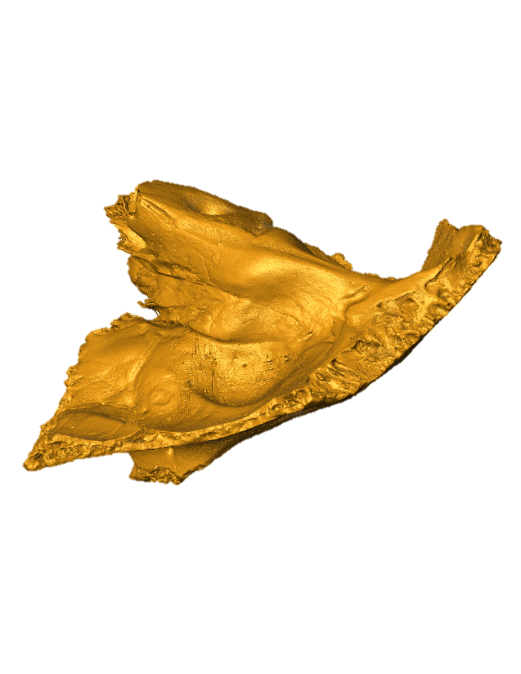

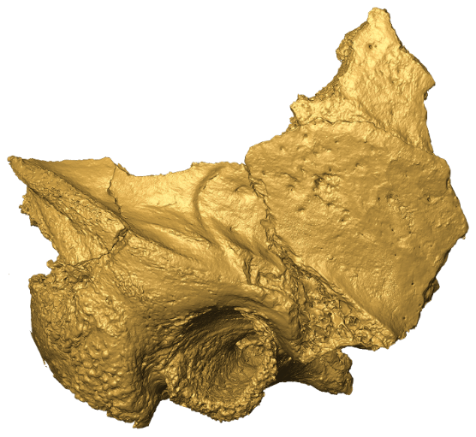

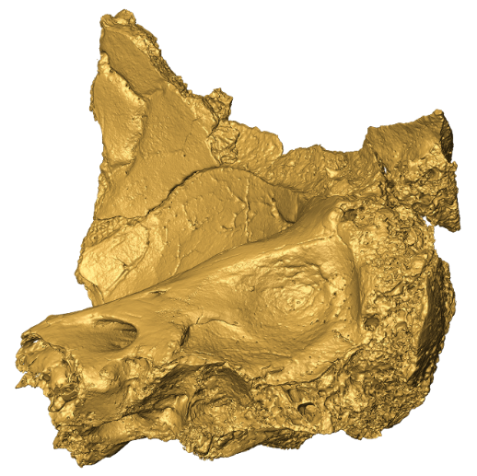

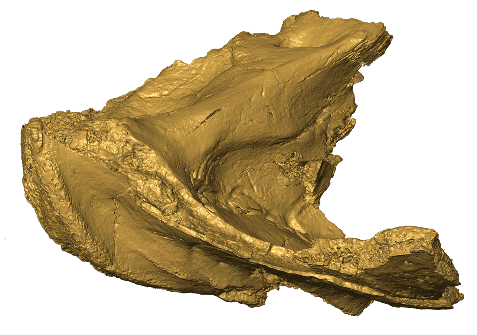


**Fig. S1.** From top to bottom: KW 9600 (right), KW 9700 (left), KW 9900 (right) and KW 10840 (left) temporal fragments (left to right: endocranial, lateral and superior views). Two scale bars: 3 cm.


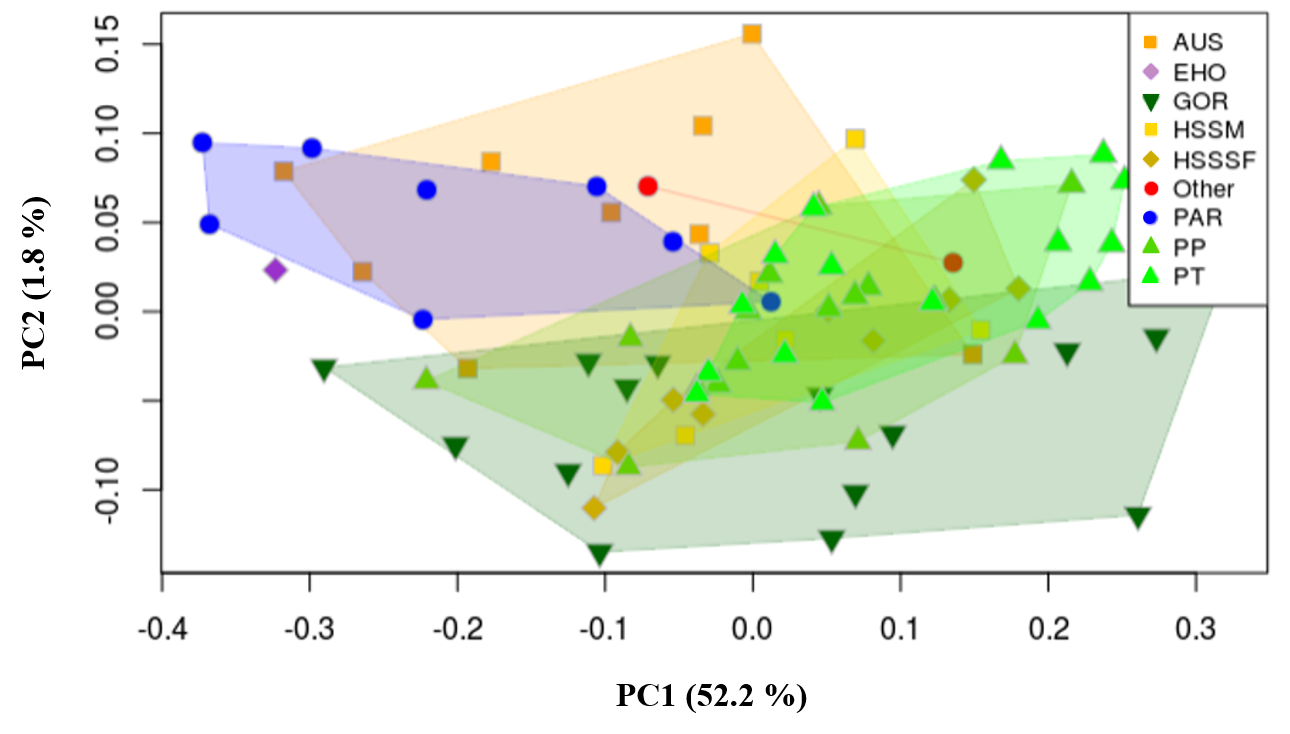


**Fig. S2.** Shape analysis of cochlear curves with geometric morphometrics. PC1 versus PC2 scatter plot obtained after PCA. Symbols: blue circles are for *P. robustus* (PAR); brown squares are for *A. africanus* (AUS); light and dark green triangles are for pygmy (PP) and common chimpanzees (PT), respectively; green inverted triangles are for gorillas (GOR); orange squares and diamonds are for female (HSSF) and male (HSSM) modern humans, respectively; violet diamond is for SK 847 (early *Homo*) (EHO); red circles are for StW 151 and StW 53 here considered as ‘unknown’ (Other).


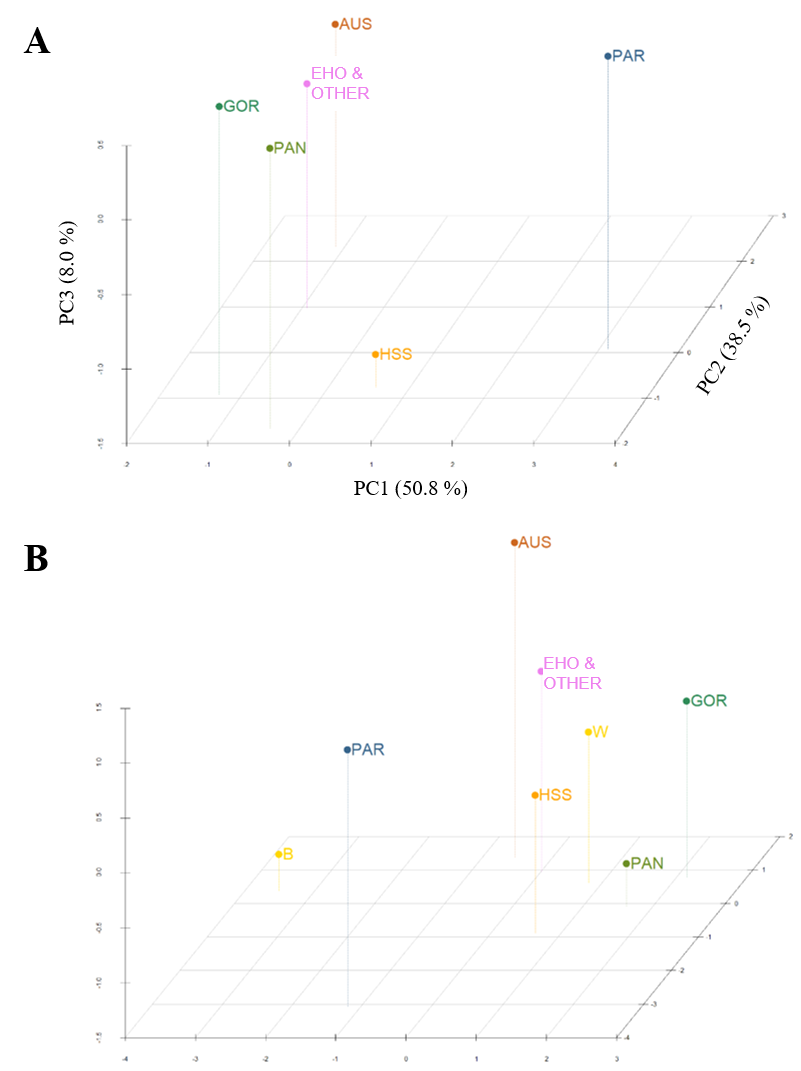


**Fig. S3.** Test for neutrality of cochlear shapes. The following six groups, and their covariance matrices, were considered: GOR, gorillas; PAN, chimpanzees; HSS, modern humans; AUS, *Australopithecus*; PAR, *Paranthropus*; OTHER, the StW 53 and StW 151 specimens here considered as ‘unknown’; EHO, SK 847 (early *Homo*). A, Principal coordinates ordination of the six covariance matrices with a scatterplot of the first three PCs. B, Heterogeneity of covariances across taxa illustrated with a principal coordinates ordination of the six covariance matrices, along with their between-group (B) and within-group (W) covariance matrices. The between-group covariance matrix (B) clearly deviates from along PC1. This allows us to reject the null hypothesis that the matrices are proportional to each other.

**
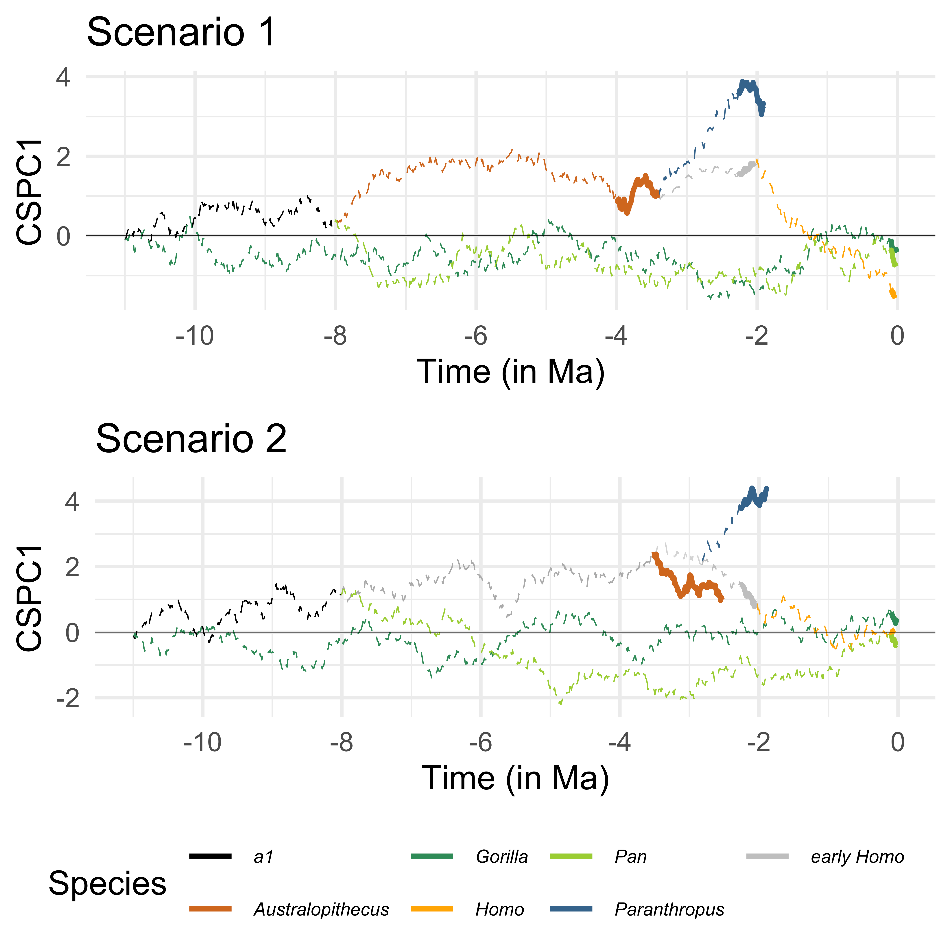
**

**Fig. S4.** Evolutionary scenarios of cochlear shapes. Two scenarios modeled as an Ornstein–Uhlenbeck (OU) process and fitted to PC1 obtained after TPCA (illustrated in Fig. 3) on a phylogeny that combines a calibration with phylogenomics, and fossil data representing *Australopithecus*, *Paranthropus*, and *early Homo* at some internal branches and nodes. “a1” represents the *Pan*/Hominin ancestor.

| **Specimen** | **Sex** | **Institution** | **Micro-CT system** | **Voxel Size** |
| --- | --- | --- | --- | --- |
| *Australopithecus* *africanus* (n=9) |  |  |  |  |
| STW 329 | Unknown | WITS | Nikon Duo | 33.1 |
| STW 498* | Unknown | WITS | Nikon Duo | 28.1 |
| STW 252/255/259* | Unknown | WITS | Nikon Duo | 33.1 |
| STW 98 | Unknown | WITS | Nikon Duo | 33.1 |
| STS 5 | Unknown | DNMNH | Metris X-Tek XT H225L | 76.15 |
| STW 578* | Unknown | WITS | Morphosource (3D Model) |  |
| STW 573* « Little Foot » | Unknown | WITS | Morphosource (3D Model) |  |
| STW 504/505* | Unknown | WITS | Nikon Duo | 30.5 |
| STS 19 | Unknown | DNMNH | Metris X-Tek XT H225L | 62.9 |
| *Paranthropus robustus* (n=8) |  |  |  |  |
| SK 879 | Unknown | DNMNH | Metris X-Tek XT H225L | 9.2 |
| SKW 18 | Unknown | WITS | Metris X-Tek XT H225L | 11.14 |
| KB 6067 | Unknown | DNMNH | Swiss Light Source, TOMCAT | 7.4 |
| KW 9600 | Unknown | WITS | Nikon Duo | 19.8 |
| KW 9700 | Unknown | WITS | Nikon Duo | 25.5 |
| KW 9900 | Unknown | WITS | Nikon Duo | 43.5 |
| KW 10840 | Unknown | WITS | Nikon Duo | 64.1 |
| DNH 22 | Unknown | WITS | Nikon Duo | 26.7 |
| Early *Homo* (n=1) |  |  |  |  |
| SK 847 | Unknown | DNMNH | Metris X-Tek XT H225L | 21.7 |
| Unknown (n=2) |  |  |  |  |
| SW 151 | Unknown | WITS | Nikon Duo | 28.3 |
| StW 53 | Unknown | WITS | Nikon Duo | 30.5 |
| *Gorilla gorilla* (n=15) |  |  |  |  |
| 73018M2 | Unknown | MRAC | XtremeCT | 41.0 |
| MHNT ZOO2011.0.5 | Unknown | MHNT | XtremeCT | 41.0 |
| 2259 | Unknown | MRAC | RX-solutions EasyTom XL duo | 32.6 |
| 12675 | Unknown | MRAC | RX-solutions EasyTom XL duo | 32.6 |
| 15351 | Unknown | MRAC | RX-solutions EasyTom XL duo | 32.6 |
| 15360 | Unknown | MRAC | RX-solutions EasyTom XL duo | 32.6 |
| 15366 | Unknown | MRAC | RX-solutions EasyTom XL duo | 32.6 |
| 15367 | Unknown | MRAC | RX-solutions EasyTom XL duo | 32.6 |
| 35274 | Male | MRAC | RX-solutions EasyTom XL duo | 32.6 |
| 804 | Male | MRAC | RX-solutions EasyTom XL duo | 110 |
| 84044M8 | Unknown | MRAC | RX-solutions EasyTom XL duo | 80.0 |
| 84044M6 | Unknown | MRAC | RX-solutions EasyTom XL duo | 105 |
| 29102 | Female | MRAC | RX-solutions EasyTom XL duo | 100 |
| 31132 | Female | MRAC | RX-solutions EasyTom XL duo | 20.0 |
| 22763 | Male | MRAC | RX-solutions EasyTom XL duo | 20.0 |
| *Homo sapiens* (n=16) |  |  |  |  |
| EMBR121 | Female | IANPS | XtremeCT | 41.0 |
| EMBR212 | Female | IANPS | XtremeCT | 41.0 |
| EMBR281 | Female | IANPS | XtremeCT | 41.0 |
| EMBR308 | Female | IANPS | XtremeCT | 41.0 |
| EMBR385 | Female | IANPS | XtremeCT | 41.0 |
| EMBR513 | Female | IANPS | XtremeCT | 41.0 |
| EMBR576 | Female | IANPS | XtremeCT | 41.0 |
| EMBR179 | Male | IANPS | XtremeCT | 41.0 |
| EMBR205 | Male | IANPS | XtremeCT | 41.0 |
| EMBR215 | Male | IANPS | XtremeCT | 41.0 |
| EMBR249 | Male | IANPS | XtremeCT | 41.0 |
| EMBR383 | Male | IANPS | XtremeCT | 41.0 |
| EMBR388 | Male | IANPS | XtremeCT | 41.0 |
| EMBR473 | Male | IANPS | XtremeCT | 41.0 |
| **Specimen** | **Sex** | **Institution** | **Micro-CT system** | **Voxel Size** |
| EMBR479 | Male | IANPS | XtremeCT | 41.0 |
| EMBR323 | Male | IANPS | XtremeCT | 41.0 |
| *Pan paniscus* (n=16) |  |  |  |  |
| 29007 | Male | MRAC | XtremeCT | 41.0 |
| 84035M6 | Unknown | MRAC | XtremeCT | 41.0 |
| 13201 | Female | MRAC | RX-solutions EasyTom XL duo | 74 |
| 15284 | Male | MRAC | RX-solutions EasyTom XL duo | 65 |
| 26989 | Female | MRAC | RX-solutions EasyTom XL duo | 65 |
| 27000 | Unknown | MRAC | RX-solutions EasyTom XL duo | 54 |
| 27002 | Female | MRAC | RX-solutions EasyTom XL duo | 65 |
| 26992 | Unknown | MRAC | RX-solutions EasyTom XL duo | 58 |
| 888 | Unknown | MRAC | RX-solutions EasyTom XL duo | 65 |
| 84036M9 | Unknown | MRAC | RX-solutions EasyTom XL duo | 65 |
| 29003 | Female | MRAC | XtremeCT | 41.0 |
| 29051 | Male | MRAC | XtremeCT | 41.0 |
| 84036M8 | Unknown | MRAC | Optiv CT160 | 8.0 |
| 15294 | Male | MRAC | RX-solutions EasyTom XL duo | 20.0 |
| 15295 | Male | MRAC | RX-solutions EasyTom XL duo | 20.0 |
| 15296 | Female | MRAC | RX-solutions EasyTom XL duo | 20.0 |
| *Pan troglodytes* (n=16) |  |  |  |  |
| 71 | Unknown | UT-PS | XtremeCT | 41.0 |
| 72 | Unknown | UT-PS | XtremeCT | 41.0 |
| 2297 | Male | MRAC | RX-solutions EasyTom XL duo | 20.0 |
| 10415 | Unknown | MRAC | RX-solutions EasyTom XL duo | 60.0 |
| 31490 | Female | MRAC | RX-solutions EasyTom XL duo | 85.0 |
| 36281 | Unknown | MRAC | RX-solutions EasyTom XL duo | 58.0 |
| 80 | Female | MRAC | RX-solutions EasyTom XL duo | 65.0 |
| 31547 | Female | MRAC | RX-solutions EasyTom XL duo | 75.0 |
| 2538 | Unknown | MRAC | XtremeCT | 41.0 |
| 7054 | Unknown | MRAC | XtremeCT | 41.0 |
| 10732 | Unknown | MRAC | XtremeCT | 41.0 |
| 44 | Unknown | UT-PS | XtremeCT | 41.0 |
| ZOO2011.0.3 | Unknown | MHNT | XtremeCT | 41.0 |
| 82032M8 | Unknown | MRAC | RX-solutions EasyTom XL duo | 84.0 |
| 15976 | Male | MRAC | RX-solutions EasyTom XL duo | 20.0 |
| 9252 | Unknown | MRAC | RX-solutions EasyTom XL duo | 20.0 |

**Table S1**. List of specimens investigated in this study. Specimens marked with* have been interpreted as representing a « second, larger-toothed species » distinct from *A. africanus* on the basis of dental and facial morphological features^16^. Institutions are: DNMNH (Ditsong National Museum of Natural History, Pretoria, South Africa; formerly Transvaal Museum), IANPS (Institut d’Anatomie Normale et Pathologique de Strasbourg, France), MHNT (Muséum d’Histoire Naturelle de Toulouse, France), MRCA (Musée Royal de l’Afrique Centrale, Tervuren, Belgium), UT-PS (Université de Toulouse, Paul Sabatier, France), WITS (Evolutionary Studies Institute, University of the Witwatersrand, Johannesburg, South Africa). Voxel size is indicated in microns.

Description Discovery, Excavator

KW 9600 R & L petrosals, part of supraorbital area and frontal squama, October 2016, L. Modise

biparietal arch with most of R & L parietals, fragment of occipital squama

KW 9700 L petrosal October 2016, B. Maisonnier

KW 9900 R temporal and associated malleus, L petrosal, R,LI^1-2^;R,L^C^;RP^3-4^,LP^4^,R,LM^2-3^, October 2016, L. Saffre

fragments of LP^3^ and LM^1^; fragments of roots

KW 10840 fragment of L maxilla with LI^1^ & LM^1^ germs; R^C^, RP^3^, L temporal bone, R petrosal, November 2017, S. Makhele

fragments of L,R parietals & occipital squama, fragment of L sphenoid greater wing,

L exoccipital

Table S2. List and brief description of the four *P. robustus* specimens preserving petrous bones and newly discovered from Unit P at Kromdraai. R, right; L, left; I, permanent incisor; C, permanent canine; P, premolar; M, permanent molar.

|  | ECL | HZL | POL | ANL | TLI | COs<LSCm | APA<LSCm | ECL/HZL | ECL/POL | ECL/ANL | HZL/POL | HZL/ANL | POL/ANL |
| --- | --- | --- | --- | --- | --- | --- | --- | --- | --- | --- | --- | --- | --- |
| early *Homo* | | | | | | | | | | | | | |
| SK 847 | 34 | 15,4 | 18,6 | 15,9 | 40(35*) | 64(68*) | 38(40*) | 2,2 | 1,8 | 2,1 | 0,8 | 1,0 | 1,2 |
| *Paranthropus robustus* | | | | | | | | | | | | | |
| Mean | 35,0 | 15,5 | 16,9 | 13,8 | 26,4 | 41,1 | 28,9 | 2,3 | 2,1 | 2,6 | 0,9 | 1,1 | 1,3 |
| SD | 3,0 | 1,3 | 2,2 | 0,7 | 5,5 | 2,0 | 4,6 | 0,1 | 0,2 | 0,3 | 0,1 | 0,1 | 0,1 |
| Kromdraai |  |  |  |  |  |  |  |  |  |  |  |  |  |
| TM 1517 | - | - | - | - | 30(29**) | 39(33**) | 31(32**) | - | - | - | - | - | - |
| KB 6067 | 31,9 | 14,8 | 13,8 | - | 21** | 41** | 36** | 2,2 | 2,3 | - | 1,1 | - | - |
| Swartkrans |  |  |  |  |  |  |  |  |  |  |  |  |  |
| SK 879 | 39,1 | 17,4 | 18,1 | 13,4 | 24(22*) | 43(44*) | 23(29*) | 2,2 | 2,2 | 2,9 | 1,0 | 1,3 | 1,4 |
| SKW 18 | 34 | 15 | 17 | 14,6 | 29 | 42 | 25 | 2,3 | 2,0 | 2,3 | 0,9 | 1,0 | 1,2 |
| SK 46 | - | - | - | - | 21* | 39* | 31* | - | - | - | - | - | - |
| SK 47 | - | - | - | - | 24* | 44* | 31* | - | - | - | - | - | - |
| Drimolen |  |  |  |  |  |  |  |  |  |  |  |  |  |
| DNH 22 | 34,9 | 14,8 | 18,8 | 13,3 | 36 | 40 | 25 | 2,4 | 1,9 | 2,6 | 0,8 | 1,1 | 1,4 |
| Kromdraai (Unit P), This study | | | | | | | | | | | | | |
| KW 9600 | 33,4 | 13,3 | 14,1 | 13,5 | 31 | 51 | 27 | 2,5 | 2,4 | 2,5 | 0,9 | 1,0 | 1,0 |
| KW 9700 | 34,8 | 14,9 | 14,2 | 14,2 | 31 | 41 | 23 | 2,3 | 2,5 | 2,5 | 1,0 | 1,0 | 1,0 |
| KW 9900 | 33,8 | 15,5 | 14,2 | 13,8 | 31 | 40 | 26 | 2,2 | 2,4 | 2,4 | 1,1 | 1,1 | 1,0 |
| KW 10840 | 33,5 | 12,8 | 14,5 | 12,6 | 29 | 52 | 35 | 2,6 | 2,3 | 2,7 | 0,9 | 1,0 | 1,2 |
|  |  |  |  |  |  |  |  |  |  |  |  |  |  |

Table S3. Measurements of the bony labyrinths of the four new specimens from Unit P at Kromdraai, a sample of South African *P. robustus* and early *Homo* specimens. Abbreviations: ECL, external cochlear length in millimeters (mm) (Braga et al. 2013); HZL, POL and ANL are the arc lengths (mm) of the horizontal, posterior and anterior semi-circular canals, respectively; TLI, transverse labyrinthine index, COs<LSCm, angle (in degrees) between the cochlear basal turn and the horizontal semi-circular canal, APA<LSCm, angle (in degrees) between the ampullar line and the horizontal semi-circular canal^47^. *data from^47^, **data from^26^.

**Supplementary References**

Braga, J., Thackeray, J.F., Bruxelles, L., Dumoncel, J. and Fourvel, J.B., 2017. Stretching the time span of hominin evolution at Kromdraai (Gauteng, South Africa): recent discoveries. *C. R. Palevol* **16**, 58-70 (2017).

Brain, C.K. The Transvaal Ape-Man-Bearing Cave Deposits (Transvaal Museum, Pretoria, 1958).

Broom, R. The Pleistocene anthropoid apes of South Africa. *Nature* **142**, 377-379 (1938).

Dryden, I.L. and Mardia, K.V. Statistical Shape Analysis (Wiley and Sons, 1998).

Bruxelles, L., Maire, R., Couzens, R., Thackeray, F. and Braga, J., 2016. A revised stratigraphy of Kromdraai. in Kromdraai, a Birthplace of *Paranthropus* in the Cradle of Humankind (eds. Braga, J. & Thackeray, J.F.) 31-47 (SunMedia Metro, Johannesburg, 2016).

Ngoloyi, M., Dumoncel, J., Thackeray, J.F. and Braga, J. A new method to evaluate 3D spatial patterns within early hominin-bearing sites. An example from Kromdraai (Gauteng Province, South Africa). *J. Archaeol. Sci. Reports* **32**, 102376 (2020).

Partridge, T.C. Some preliminary observations on the stratigraphy and sedimentology of the Kromdraai B hominid site. *Palaeoecology of Africa and the Surrounding Islands* *15*, 3-12 (1982).

Samir, C. and Adouani, I. C1 interpolating bézier path on riemannian manifolds, with applications to 3D shape space. *App. Math. Comput*. **348**, 371-384 (2019).

Samir, C., Loubes, J.M., Yao, A. and Bachoc, F. Learning a gaussian process model on the Riemannian manifold of non-decreasing distribution functions. in Trends in Artificial Intelligence, Lecture Notes in Computer Science (eds. Nayak, A.C. and Sharma, A.) 107-120 (Springer, 2019).

Vrba, E. The Kromdraai australopithecine site revisited in 1980: recent investigations and results. *Ann. Transv. Mus*. **33**, 17-60 (1981).
